# Direct Evidence for a Carbon–Carbon One‐Electron σ‐Bond, or a Weak Carbon–Carbon Interaction?

**DOI:** 10.1002/open.70242

**Published:** 2026-06-08

**Authors:** Costantino Zazza, Felice Grandinetti, Nico Sanna, Stefano Borocci, Henry S. Rzepa

**Affiliations:** ^1^ Department for Innovation in Biological Agro‐food and Forest systems Università della Tuscia (DIBAF) Viterbo Italy; ^2^ Istituto per i Sistemi Biologici del CNR (ISB) Sede di Roma ‐ Meccanismi di Reazione c/o Dipartimento di Chimica Sapienza Università di Roma Rome Italy; ^3^ CNR‐ISTP (Istituto per la Scienza e Tecnologia dei Plasmi) Bari Italy; ^4^ Department of Chemistry Imperial College London London UK

**Keywords:** chemical bonding, computational methods, density functional theory, quantum theory of atoms in molecules, Raman spectroscopy

## Abstract

Shimajiri et al. (*Nature*, **2024**, 634, 347–351) reported for the first time on direct evidence for a carbon–carbon one‐electron σ bond in a recently synthesized stable radical cation. Single‐crystal X‐ray diffraction analysis, electron density maps, and RAM spectroscopy provided analytical results supporting the derived picture. However, the theoretical counterpart at the density functional theory level tends to support a different viewpoint, namely two sp^2^ carbon atoms at a rather high distance of 2.921(3) Å undergoing a weak non‐covalent interaction. In this contribution, we wish to highlight the discrepancies observed in relation to laboratory measurements as well as the similarities with a recent research by L. I. Lugo‐Fuentes (*Phys. Chem. Chem. Phys.*, **2026**, 28, 683–691) with the aim of promoting new theoretical studies—going beyond the Kohn–Sham (KS) framework in the field of wavefunction correlated methods—on what could become a new benchmark system for the coming years. Taken together, the available data do not unequivocally demonstrate the existence of a discrete C─C one‐electron σ‐bond but instead highlight the difficulty of distinguishing extremely weak bonding interactions from non‐bonded or delocalized radical states.

## Graphical abstract

Experiments reporting the existence of a single‐electron C─C σ bond in a synthesized stable radical cation open up new perspectives in the study of the existence of chemical bonds. However, the supporting theoretical results at density functional theory cost do not seem to fully support this evidence. We highlight the discrepancies observed to promote new theoretical studies on what could become a new benchmark system for the coming years.

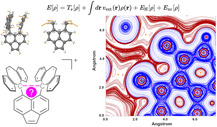

## 1 The Carbon–Carbon One‐Electron σ‐Bond

More than 100 years ago, before the quantum mechanical treatment of molecules had been formulated, G. N. Lewis proposed [1] a simple model for chemical bonding that is still taught today. This is the idea of the three categories of bond we know as single, double and triple, comprising respectively two, four, and six shared electrons each, at least for the very common carbon–carbon bond. A little more than a decade ago, this was extended upwards to the eight‐electron quadruple bond [2]. Now, at the other extreme of downwards, a molecule has been characterized in the solid state with a claimed one‐electron C─C bond [3]. More specifically, Shimajiri et al. [3] reported the isolation of a stable radical cation—a hexaphenylethane (HPE) derivative featuring two spiro‐dibenzocycloheptatriene (DBCHT) units. Based on experiments and theoretical simulations, they concluded these presented “direct evidence for a carbon–carbon one‐electron *σ*‐bond”. Such a bonding motif finds its origins in the work by Linus Pauling [4], who postulated it for the exemplary case of the H_2_^•+^ radical cation. After carefully considering the evidence on which the authors [3] based their conclusions, at least at the theoretical level, we do not believe that their conclusions are so definitive and sound as to provide direct evidence for a carbon–carbon one‐electron *σ*‐bond, within the proposed density functional theory (DFT) framework. We will base our conclusion not on new data but simply on a different interpretation of the evidence provided by the authors.

The investigated systems include three species related by redox reactions, namely the neutral **1**, the radical cation **1** ^•+^ and the dication **1** ^2+^ reported in Figure 2 of the original paper [3]. One of the seven‐membered DBCHT moiety in **1** ^•+^ adopts a bent unsymmetric geometry while the other facing DBCHT unit exhibits an almost planar geometry. Despite the unsymmetrical bent‐planar geometry, the sum of the three bond angles around the C_1_ and C_2_ atoms suggested their sp^2^ hybridization. From the X‐ray analysis of **1** ^•+^I_3_^−^, electron density maps (i.e., Fo‐Fc by difference) estimated the residual density between the central C_1_ and C_2_ atoms. More specifically, two independent X‐ray measurements on different single‐crystalline samples of **1** ^•+^ acquired at 100K confirm the reproducibility of this observation: a residual electronic density well confined within the C_1_ and C_2_ direction, with a contour level of 0.05 Å^−3^ assigned to a bonding between C_1_ and C_2_. Such a magnitude is comparable to that of carbon–carbon bonds within the neighboring naphthalene group [3]. The electron sharing between DBCHT units was then attributed to the formation of a C•C one‐electron σ‐bond promoted by the oxidation processes. To obtain more information on the strength of their proposed C•C one‐electron σ‐bond, the authors explored the Raman spectrum of a single crystal of **1** ^•+^I_3_^−^, and noticed a normal mode at 379 cm^−1^ that was assigned as the stretching of the proposed C_1_‐C_2_ single‐electron covalent bond.

**FIGURE 1:**
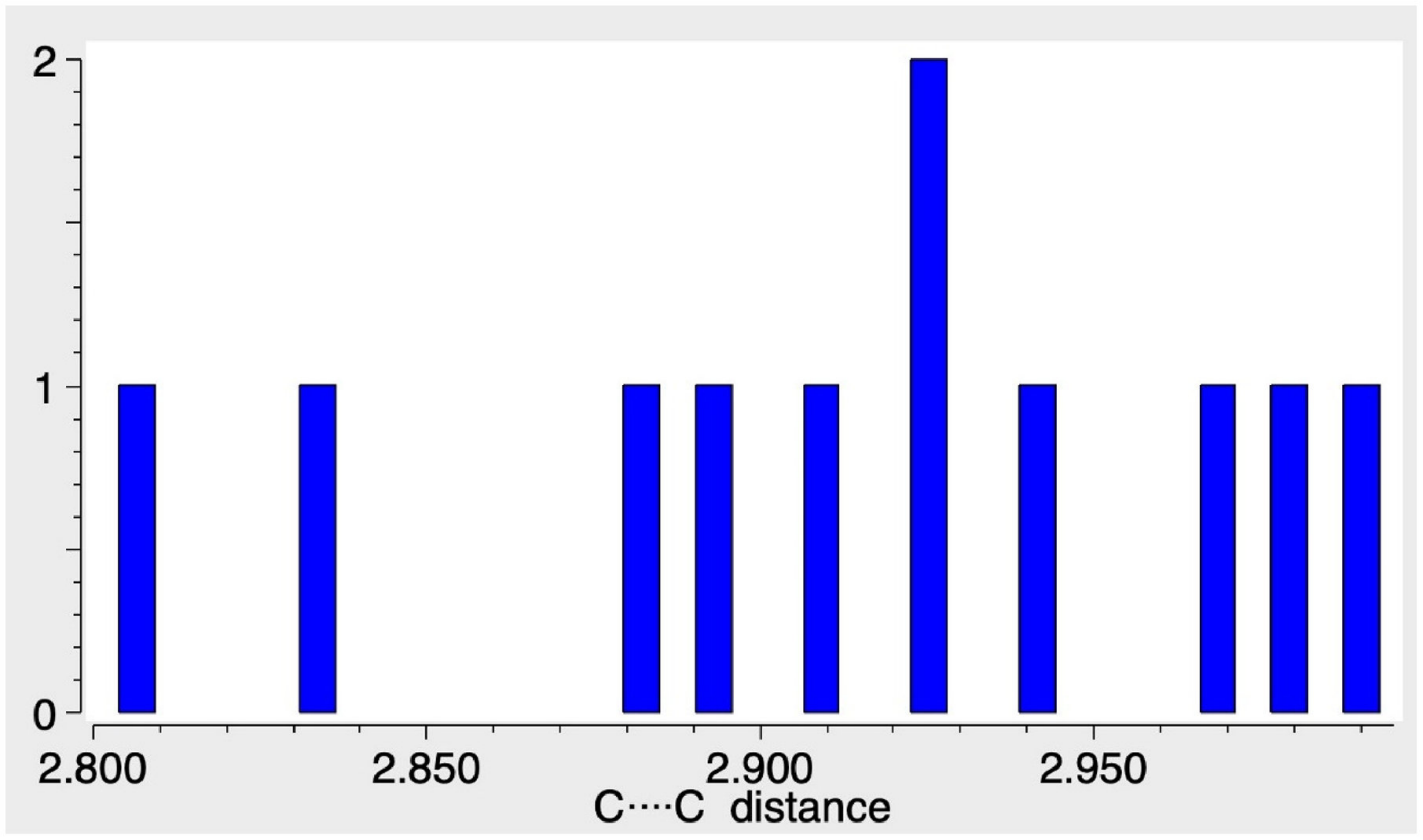
Distribution of C─C bond lengths (Å) found in the CSD [5] for mono and di‐cationic species in the region 2.8–3.0 Å.

**FIGURE 2:**
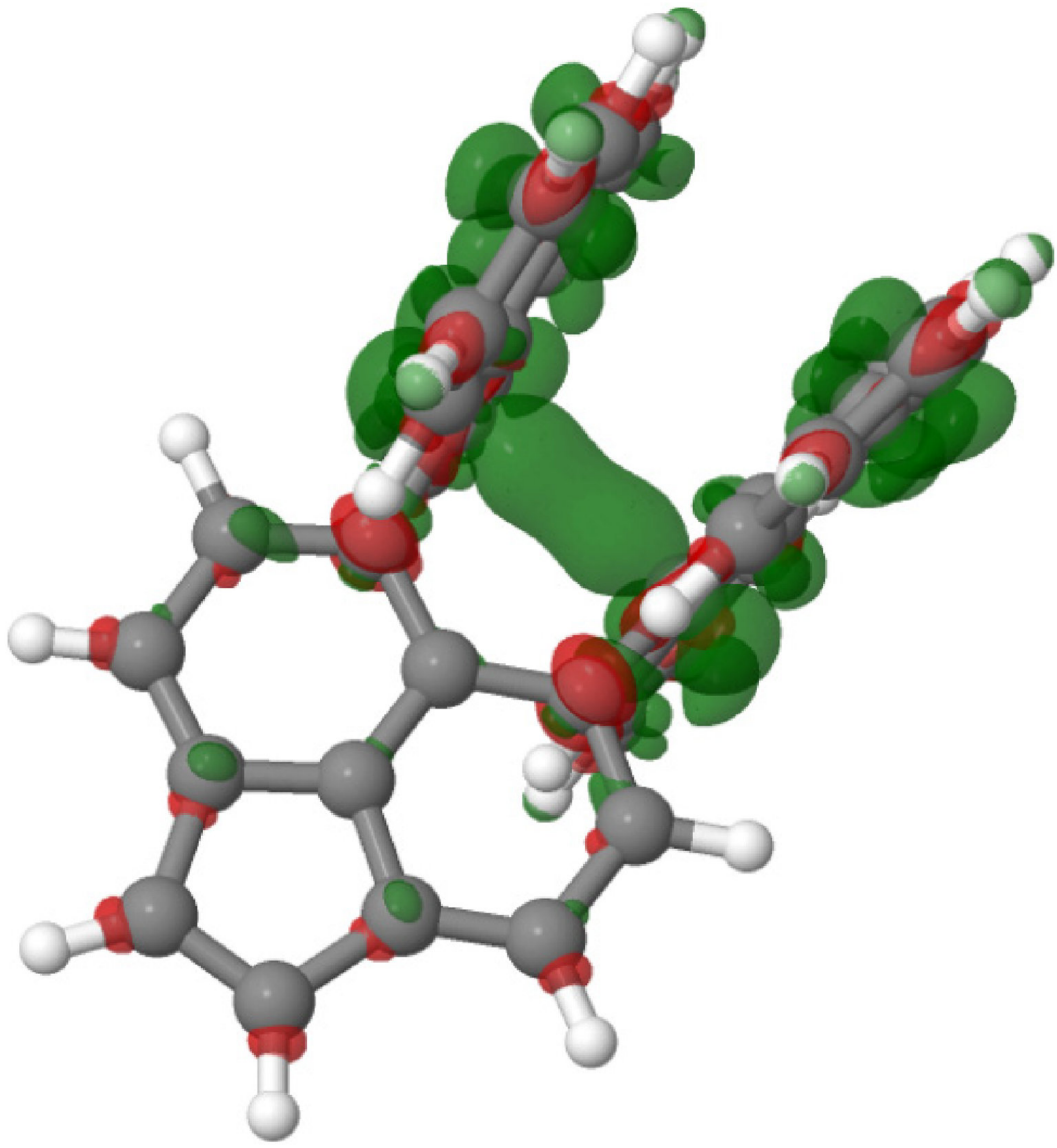
ωB97XD/Def2‐TZVPP electron density difference map (isosurface value 0.002 au) obtained by subtracting the computed density of the radical cation **1** ^•+^, from that of the dication, **1** ^2+^, at the geometry of the former species. Given the sign imposed, the electronic density moves from the green to the red areas.

With this information in mind, a search of the crystal structure database (CSD) [5] was performed with the aim of extracting the longest central C─C bonds presumably responsible for the one‐electron σ‐bond. We considered the two selected carbon atoms as triple coordination with the fourth bond imposed as variable and with values ≥ 2.8 Å. This resulted in 10 hits shown in Figure 1, all revealed as dications, with the central C─C distance ranging from 2.8 to 3.0 Å. So the unique feature of the research published by Shimajiri et al. is that they were able to find a system where oxidation did not proceed directly to the dication, but stopped at the 1‐electron level to give a radical cation instead. The same authors [3] also made the dication, and they report a C─C length of 3.03 Å for this species, broadly in accord with the range shown in Figure 1 and a reduced value of 2.92 Å for the radical cation (Δ*r*_C–C_ 0.11 Å). This is quite a small contraction induced by the formation of the one–electron bond, which is already hinting that it might actually be a particularly weak bond. Moreover, in addition to previous results, we proceeded by performing DFT calculations [6] on these species, at the ωB97XD/Def2‐TZVPP level [7, 8, 9]. At this level, the di‐ and monocationic C─C bond lengths came out as 3.075 and 2.867 Å (Δ*r*_C–C_ 0.21 Å), a slightly larger contraction than that reported, but still representing a weak bond. With wavefunctions now available for these species, we decided to inspect the electron densities. This was calculated at the geometry of the radical cation. At the same geometry, the dication was calculated and the two electron densities subtracted. The resulting density surface by difference, representing one electron, is shown in Figure 2. As expected, the most significant feature occurs in the C─C region, but quite a lot of this one electron is also distributed around the aromatic rings. So already we see that this “1‐electron” bond is in fact only a fraction of one electron—again an indication that it is a weak bond.

Noncovalent interactions (NCIs)—including hydrogen bonding, van der Waals forces, π–π stacking, halogen bonding, and steric repulsion—play a central role in determining molecular structure, reactivity, supramolecular organization, and biomolecular function. Although individually weaker than covalent bonds, their cumulative effects govern phenomena such as protein folding, molecular recognition, crystal packing, and catalytic selectivity. A robust and chemically intuitive framework for identifying and visualizing these interactions is therefore essential in many research fields. The Noncovalent Interaction (NCI) method, originally introduced by Erin R. Johnson and Weitao Yang [10], provides a real‐space approach based exclusively on the electron density (ρ) and its derivatives. Unlike energy decomposition analysis or perturbative methods, NCI does not require prior definition of interacting fragments. Instead, it exploits features of the electron density topology to reveal regions associated with weak interactions in a molecule or between molecular fragments.

**FIGURE 3:**
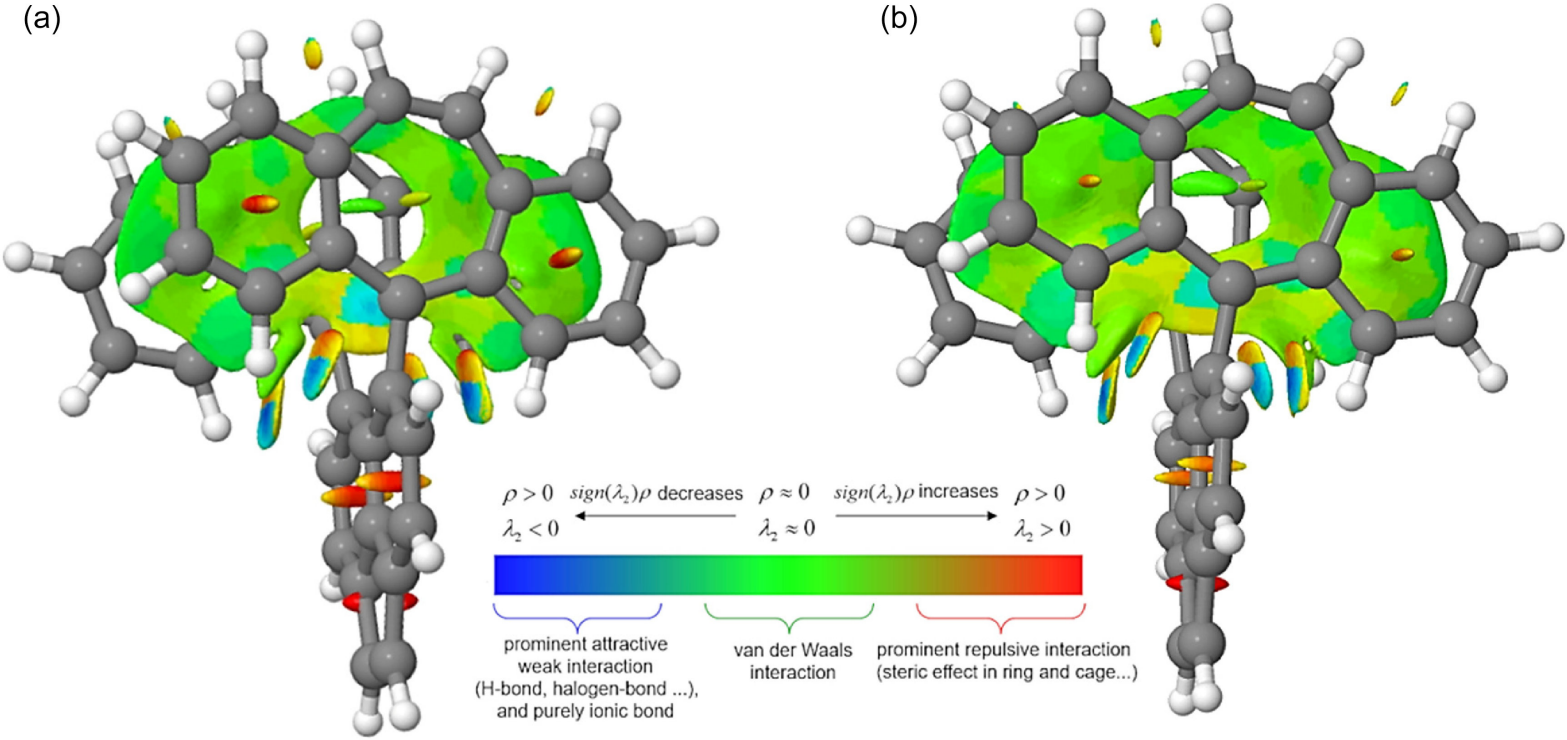
(a) 3D Non‐Covalent Interactions (NCIs, the parameters used are those reported in the original article [10]), characterizing the gaseous radical cation, **1** ^•+^, at ωB97XD/Def2‐TZVPP level of computation. (b) The same as (a) but for the dication form, **1** ^2+^.

The resulting NCI analysis is shown in Figure 3 for both the radical cation and the di‐cation at the same geometry. Plotting the Reduced Density Gradient (RDG) against *sign*(*λ*_2_)*ρ*—with *λ*_2_ corresponding to the second eigenvalue of the Hessian matrix of the electronic density—produces characteristic spikes that classify interaction types. The color coding in the NCI surface analysis in Figure 3 means that dark blue indicates strong non‐covalent interactions such as hydrogen bonds, paler blue or cyan areas are weaker ones and green is weaker still and typical of π–π stacking regions rather than bonds between two atoms. These are all deemed stabilizing, whereas orange and red regions denote steric repulsions. Enclosed by two of the cyan regions are dark blue ones indicating attractive noncovalent aryl C─H/π interactions between the DBCHT units and the local six‐membered aromatic regions of the stopper moiety, whilst the third cyan region, which is localized between the DBCHT substituents—in a π–π parallel stacking regions, contains only a small blue part. This third cyan region is indeed in the hypothesized C_1_─C_2_ one‐electron bond region, but using this analysis it clearly emerges as only a “weak” noncovalent interaction [11]. Most importantly, the observed trend results are in line with a very recent investigation [12] showing the same interaction character between the *ipso* carbon atoms (C_1_ and C_2_) within the facing DBCHT units irrespective of the presence of the **I**_**3**_^**‐**^ counterion. The two sets of authors independently converged in their conclusions to show that the C_1_–C_2_ interaction is weak, highly delocalized, and governed by subtle electronic effects rather than a single‐electron *σ*‐bond.

**FIGURE 4:**
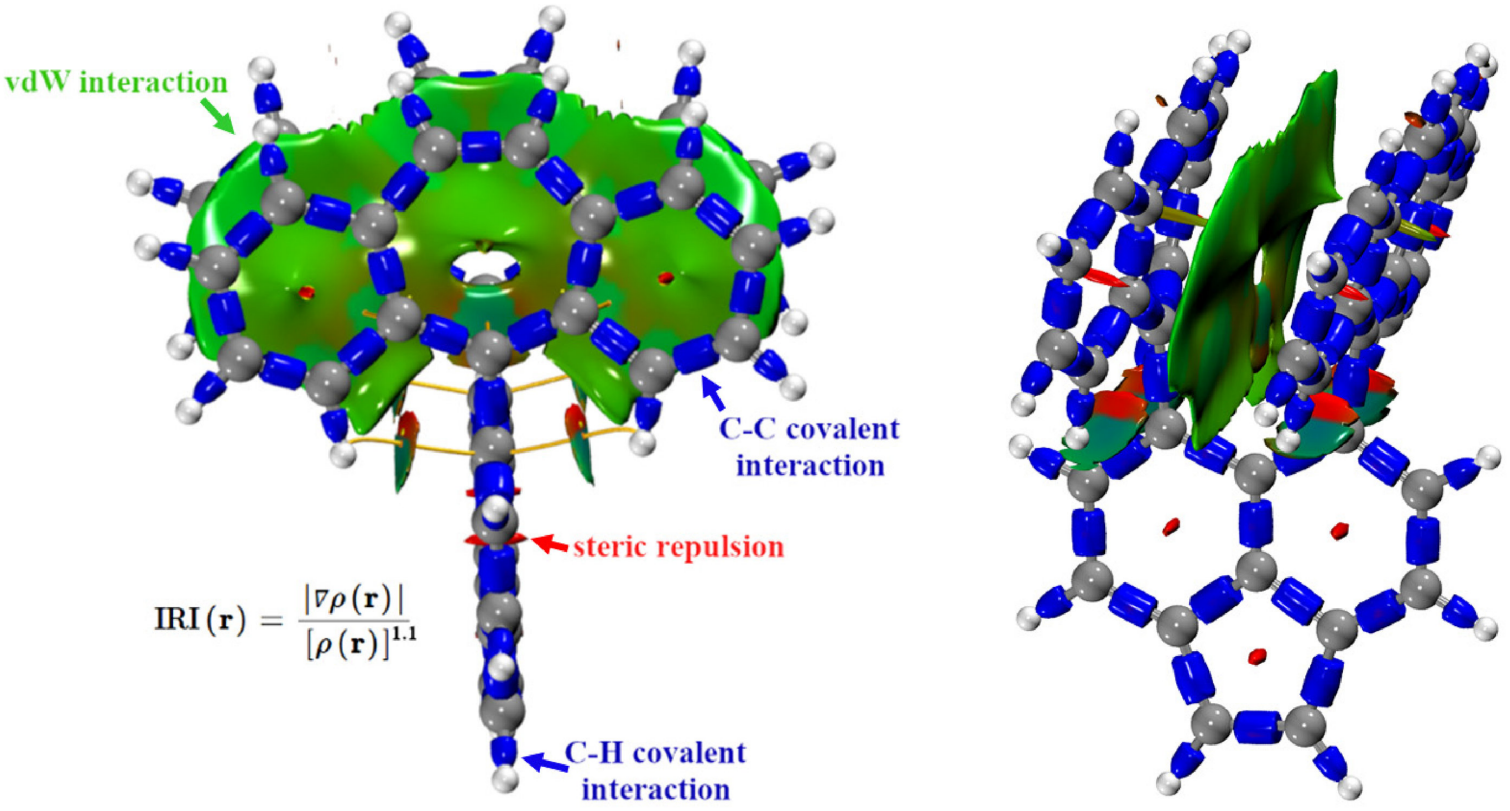
Isosurface maps of IRI = 1.0 for the **1** ^•+^ molecular system at UM06‐2X/6‐311+G** level of computation. *sign*(*λ*_2_)*ρ* is mapped on the isosurfaces according to coloring method of Figure 3. Grid spacing is 0.12 *a*_0_.

This scenario is further reinforced by deriving the Interaction Region Indicator (IRI) [13], representing a real‐space descriptor revealing both chemical bonds and weak interactions over the eclipsed form of the **1** ^•+^ in the optimized geometry reported by Shimajiri et al. [3]. The IRI plotting script provided by Multiwfn toolbox [14] is used, and the graphical results are displayed in Figure 4. Isosurface maps of IRI = 1.0 well reveal regions corresponding to each covalent bond, and noncovalent forces arising from steric repulsions and vdW interactions can also be clearly visualized. The vdW interaction region between the two parallel aromatic DBCHT moieties of **1** ^•+^ is also clearly revealed by the corresponding IRI isosurfaces; the weak interaction due to the close contact between the *ipso* C_1_‐C_2_ carbon atoms can also be detected from this analysis. We might conclude from this inspection of the reported molecule containing a one‐electron C─C bond [3], that it probably belongs to the class known as an “interaction” rather than an actual bond. Even as an interaction, it is not particularly strong – in part this is probably because only a fraction of that one electron is actually located in the C─C local region, with the rest being distributed around the aromatic rings.

**FIGURE 5:**
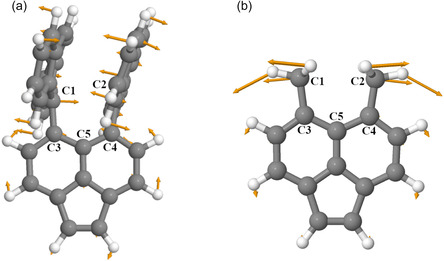
(a) UM06‐2X/6‐311+G** normal vibrational mode of the gaseous **1** ^•+^ associated with the eigenvalue at 390 cm^−1^. (b) UM06‐2X/6‐311+G** normal vibrational mode of a model system with methyl groups replacing the DBCHT moieties of **1** ^•+^. The corresponding eigenvalue is at 331 cm^−1^.

Moreover, we would like to stress the fact that the normal vibrational mode motion experimentally detected in the original article is best described as a bending rather than as a stretching mode. In fact, as shown in Figure 5 and as detailed in the animation provided in the supplementary material, the derived atomic vectors of this normal mode, theoretically predicted at 390 cm^−1^, point to a C_3_,C_4_,C_5_ bending with a constant angle between the C_2_,C_4_,C_5_ and C_1_,C_3_,C_5_ moieties. Similar results are also obtained for a model system in which the DBCHT moieties are actually replaced by two methyl groups. The vectors of the normal mode predicted at 331 cm^−1^ show again a diffuse bending motion with a clear contribution of the C_3_,C_4_,C_5_ bond angle. In the presence of a stretching component with a prominent C_1_,C_2_ contribution, these underlying angles should be modulated in a way that follows the mutual interatomic distances along the corresponding QTAIM Bond Path (BP) [15, 16]. This modulation is not observed since these pairs of angles move as rigid bodies.

**FIGURE 6:**
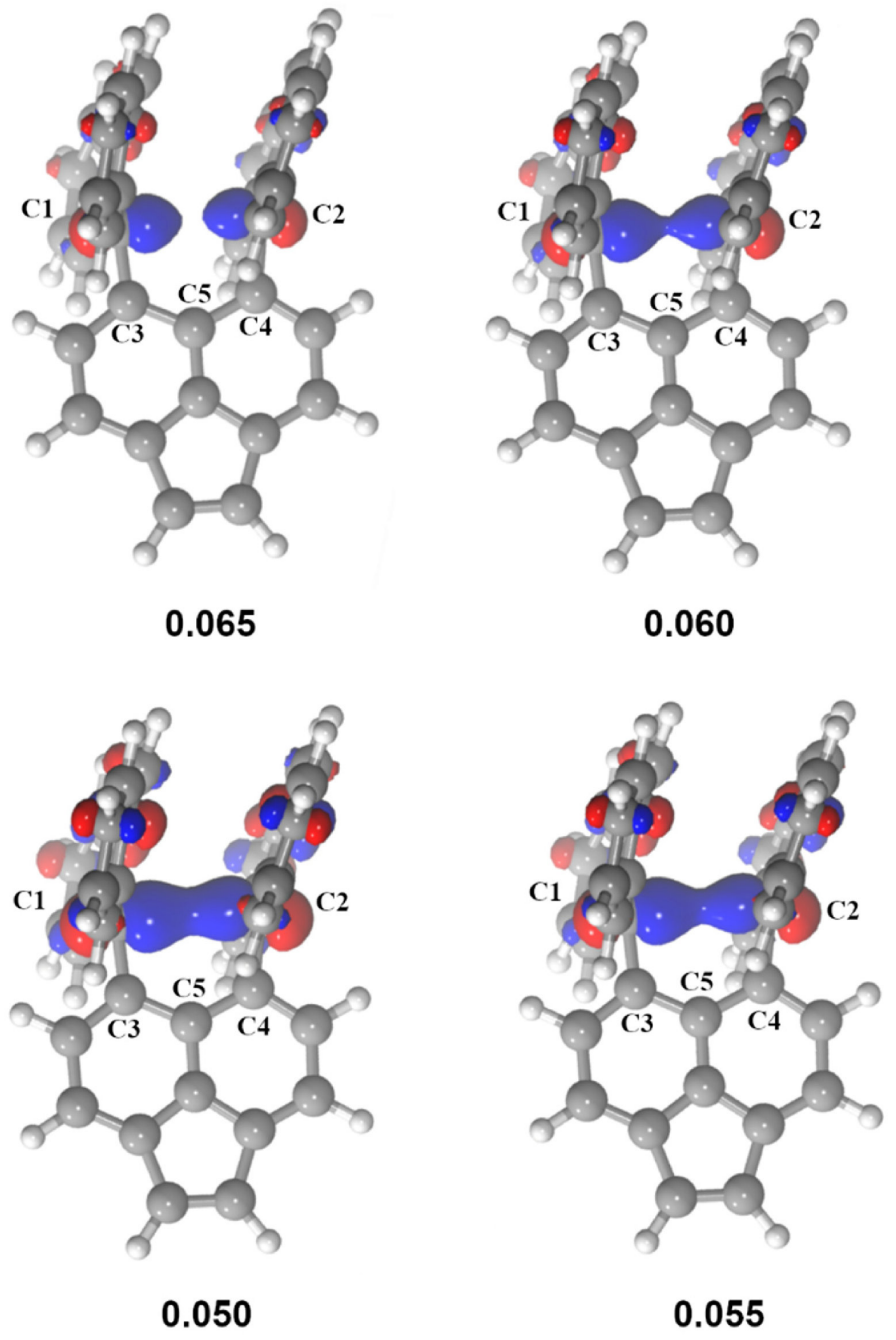
UM06‐2X/6‐311+G** α‐SOMO molecular orbital for the optimized eclipsed form of gaseous **1** ^•+^. The positive (+) part of the corresponding eigenvector is in blue, and the quoted isodensity values are in *e*·*a*_0_^−3^.

Always remaining in this context, the results of the theoretical bonding analysis also cast doubt on the proposed C•C one‐electron σ‐bond as remarked by J. Barroso‐Flores and co‐workers [12]. Shimajiri et al. [3] plotted the Kohn–Sham orbitals of **1** ^•+^, particularly the *α*‐SOMO and *α*‐LUMO. Using an isovalue of 0.050 *e·a*_0_^−3^ and looking at the plotted shape, they concluded these two eigenvectors are representative of *σ* and σ*‐type bonding/antibonding orbitals. They also reported the NBO composition of the *σ*‐type orbital (2*s* 0.2%–0.3% and 2*p* 99.6%–99.7%) hosting on‐average 0.76 electrons. A Foster‐Boys analysis also revealed the dominant contribution of C_1_(28%) and C_2_(27.5%), with minor contributions, if any, from the other atoms. However, the shape of the *α*‐SOMO is somewhat unusual for a typical covalent bond. We would have in fact expected most of the electron density to be localized at the midpoint between the two C interacting atoms. The plotted orbital, shows instead a pronounced concavity at the middle of the QTAIM BP. Thus, using the geometry of the eclipsed form [3], we plotted the *α*‐SOMO using isovalues different from 0.050 *e·a*_0_^−3^. As shown in Figure 6, the shape of the orbital is actually strongly sensitive to the used isovalue, and the comparison of the various plots best reveals its prominent identity: two 2*p* atomic orbitals localized at C_1_/C_2_ but unable to share the single electron due to the large distance between the atoms. As a matter of fact, no increase of the negative charge is observed by increasing the isovalue chosen for the plot. Such a residual and localized electronic density is, indeed, peculiar of covalent – or partially covalent – bonds [1, 4, 17]. The spin density also features the same trend, although with different isovalues (see also the data reported in Figure 5 in Ref [12].

In addition, as also noted by the same authors [3], at the Bond Critical Point (BCP) between C_1_ and C_2_, irrespective of the used X‐ray/optimized DFT structures, the local energy density *H*(***r***_**cp**_) is positive with values of 0.00057/0.00044 hartree·*a*_0_^−3^ and is typical of non‐covalent contacts of variable nature. The rather low values of the *ρ*(***r***_**cp**_), 0.01392/0.01461 *e*·*a*_0_^−3^, and the positive values of its Laplacian are also in line with this assignment. While the authors suggested an explanation for the positive value of the *H*(***r***_**cp**_) [18], the actual bonding situation is best perceived by looking at the 2D and 1D plots of the *H*(***r***) over the C_1_‐C_2_‐C_5_ plane and along the path connecting C_1_ and C_2_, respectively.

**FIGURE 7:**
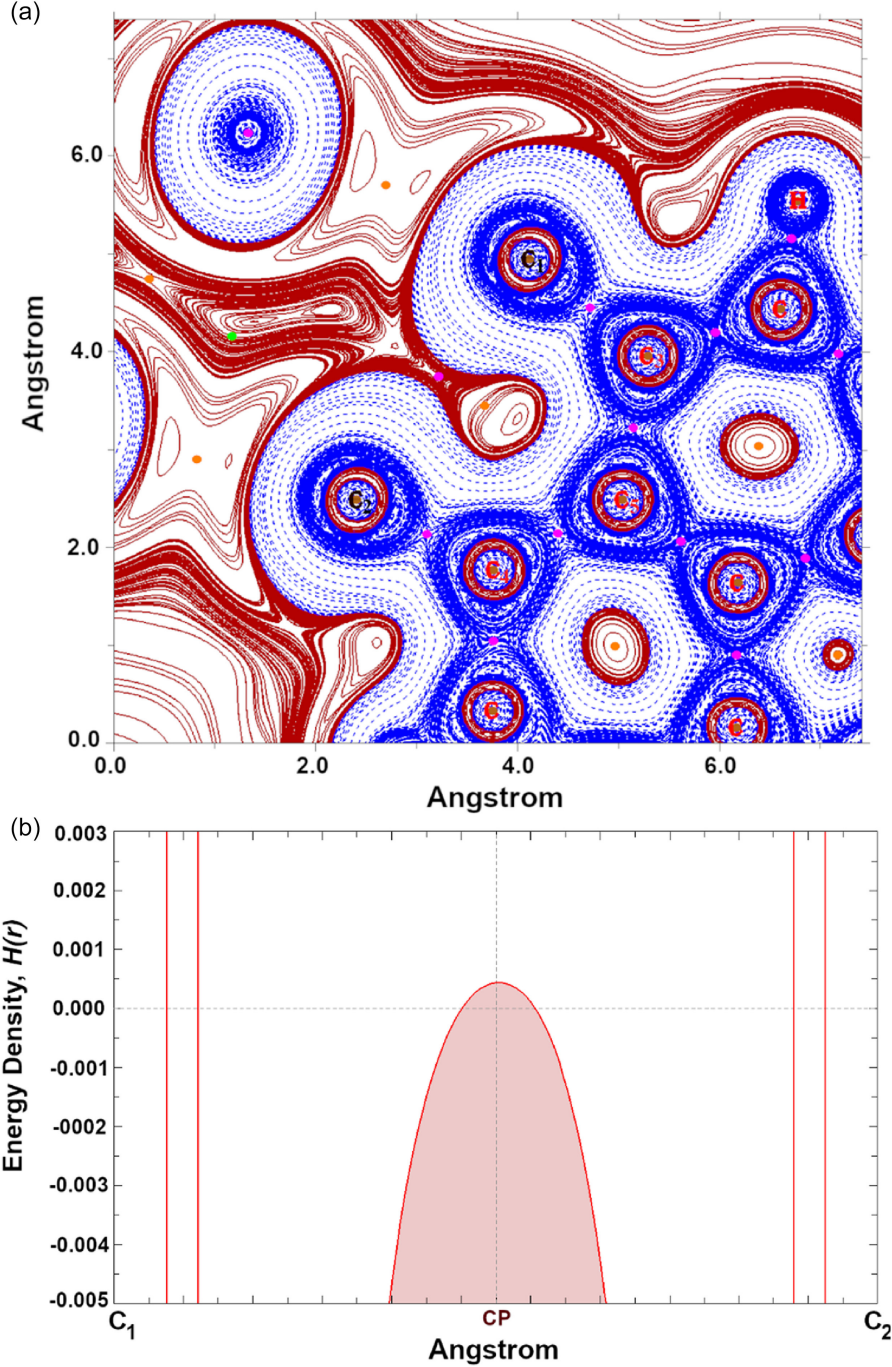
(a) 2D plot of the *H*(***r***) of the gaseous **1** ^•+^ (at the UM06‐2X/6‐311+G** level) in the plane defined by C_1_, C_2_ (in black) and C_5_ (in red). Solid/brown and dashed/blue lines correspond, respectively, to positive and negative values. The dots in magenta indicate the CPs (+3,−1). (b) 1D plot of the *H*(***r***) (hartree·*a*_0_^−3^) along the BP connecting C_1_ and C_2_.

The BCP along the C_1_‐C_2_ path (Figure 7a) falls in a positive region of *H*(***r***), and the latter features a maximum of 0.00044 hartree·*a*_0_^−3^ along the BP (Figure 7b). In general, as already discussed in previous papers [19, 20, 21, 22], this function partitions the atomic space into inner regions featuring negative values, indicated as *H* ^‐^(***r***), and outer regions of positive values, indicated as *H* ^*+*^(***r***). When two interacting atoms form a chemical bond, these regions combine in ways that signals the nature of the interaction. As shown in Figure 7a, the two C atoms overlap part of their *H* ^*+*^(***r***) regions, their *H* ^‐^(***r***) domains remaining closed and separated by a region of positive values of *H*(***r***): this suggests weak non‐covalent interactions. This bonding character becomes even more clear by inspecting the different nature of the C_1_‐C_3_ and C_2_‐C_4_ interactions. In fact, as shown in Figure 7a, as observed for typical covalent bonds, the corresponding BPs are plunged in a continuous region of negative *H*(***r***) values [23].

**FIGURE 8:**
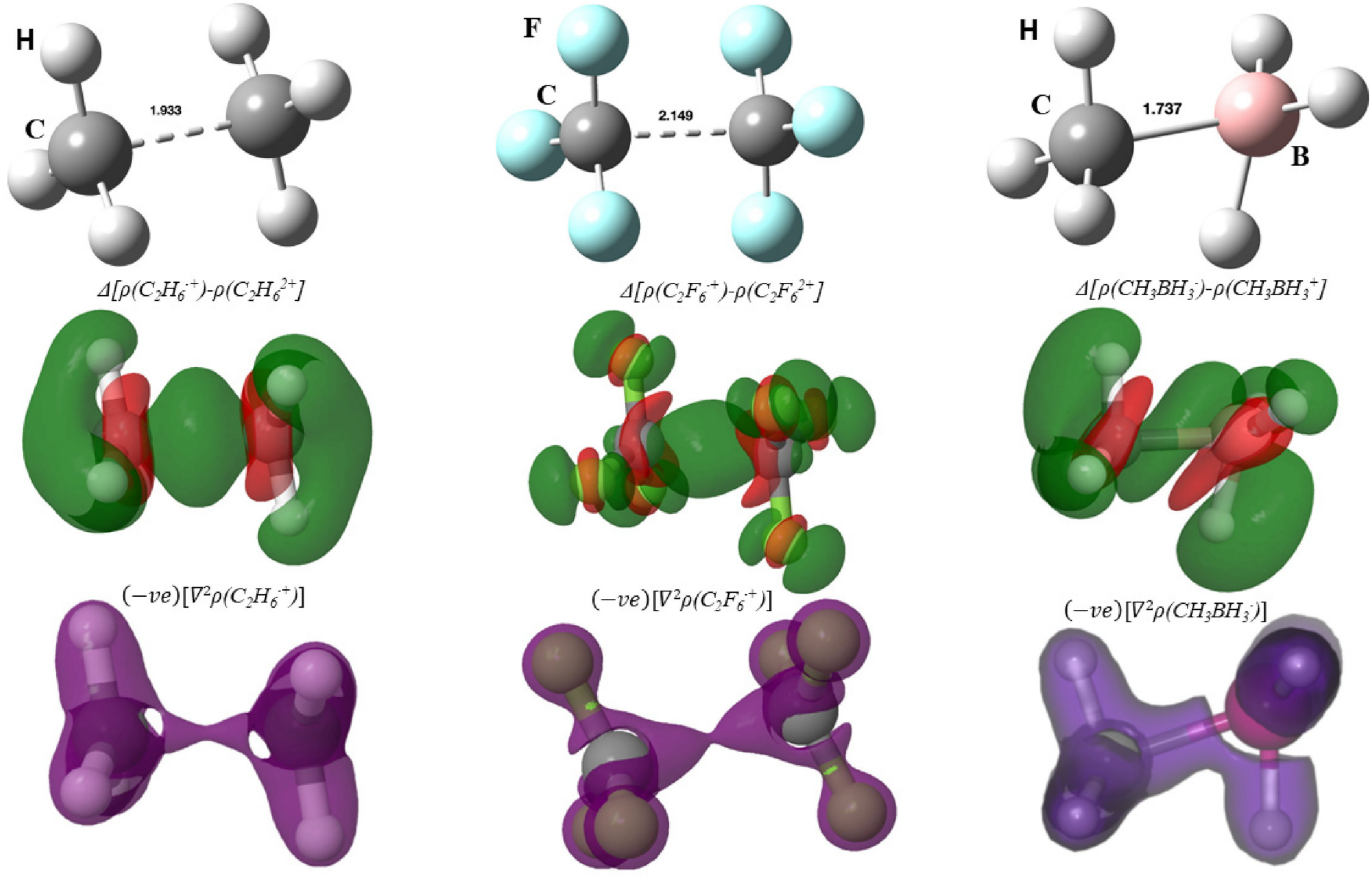
Upper panel: wB97XD/Def2‐TZVPP geometries of gaseous C_2_H_6_^•+^, C_2_F_6_^•+^, and CH_3_BH_3_^•^. Middle panel: electron density difference map, obtained by subtracting the computed density of the dication from that of the radical cation at the geometry of the former for the C_2_H_6_^•+^ and C_2_F_6_^•+^; for the CH_3_BH_3_^•^, the difference is between its cation at the geometry of the radical form. Given the sign imposed, the electronic density moves from green to red areas. Lower panel: ‐ve of the Laplacian of the electronic density for the C_2_H_6_^•+^, C_2_F_6_^•+^ (isovalue −0.001 *e·a*_0_^−5^) and CH_3_BH_3_^•^ (isovalue −0.04 *e·a*_0_^−5^) systems, respectively.

One of us (HSR) also recently studied the ionized forms of several molecules in gaseous‐phase condition, with the aim of revealing analytical descriptors capable of characterizing one electron σ‐bonds versus noncovalent interaction patterns [24, 25]. Three illustrative examples are displayed in Figure 8: ethane and hexafluoroethane as radical cations, and methyl‐λ1‐borane as a neutral radical, being isoelectronic with ethane radical cation. In the upper panel of this figure, the optimized geometry of C_2_H_6_^•+^, C_2_F_6_^•+^, and CH_3_BH_3_^•^ molecular systems are reported, and the mutual distance in Å between C─C and C─B is also shown. The C_2_H_6_^•+^ species investigated features a C─C length of 1.933 Å with a C─C stretching vibrational frequency of 477 cm^−1^ [24]. Could we consider these values as indicative of the presence of a single C─C bond? To reply to this question, the normal mode analysis was supported by an electronic density difference plot (Δ[*ρ*(C_2_H_6_^•+^)‐*ρ*(C_2_H_6_^2+^]) and a 3D representation of the Laplacian of the electronic density as extracted from the derived C_2_H_6_^•+^ converged electronic wavefunction. As can be clearly observed, the density difference between neutral ethane and its radical cation— at the geometry of the former—suggests that the electron has been removed from the C─C region accompanying the formation of the vertical radical cation. The Laplacian of the electronic density contoured for negative values of this function ‐ isosurface −0.001 *e·a*_0_^−5^ ‐ show a (small) negative value along the C─C region that still results in a diagnostic for the presence of a one‐electron σ‐bond in the C_2_H_6_^•+^ system. A similar trend was also observed for the C_2_F_6_^•+^ species [24]. More specifically, in this specific case, the two‐electron C─F σ‐bonds are much lower in energy than the C─C bond, so when the molecule is ionized, an electron escapes from the σ‐C─C bond rather than any of the C─F bonds. At the ωB97XD/Def2‐TZVPP level, a much shorter C─C bond of 2.149 Å with respect to that reported by Shimajiri et al. [3] is observed, with a C─C stretching vibrational frequency of 179 cm^−1^. The associated Δ[*ρ*(C_2_F_6_^•+^)‐*ρ*(C_2_F_6_^2+^] map ‐ always obtained by subtracting the computed density of the dication from that of the radical cation at the geometry of the former—confirms that an electron has been removed from the C─C region, with a smaller removal also from the C─F bonds. In addition, the $(-ve)[\nabla^{2}$ *ρ*(C_2_F_6_^•+^)] supports the sharing of one‐electron in the local interatomic C─C region. The radical CH_3_BH_3_^•^ form exhibits a B─C bond of 1.737 Å, and again, the Δ[*ρ*(CH_3_BH_3_^•^)‐*ρ*(CH_3_BH_3_^+^)] and $(-ve)[\nabla^{2}$ *ρ*(CH_3_BH_3_^•^)] evidences the one‐electron radical originates, at least in part, in the B─C bond (see Figure 8). Moreover, such a methyl‐λ1‐borane radical presented a B─C stretching vibration corresponding to 494 cm^−1^, a Wiberg bond order of 0.660, and Wiberg bond index totals of 3.51 for carbon and 3.28 for boron [25]. These can all be reasonably interpreted as a one‐electron “half” bond between C and B. With a computed bond length of 1.737 Å, it was suggested that it represents the shortest “one electron” bond thus far identified. As further evidence to what was noted, in the present contribution we have re‐processed both C_2_F_6_^•+^ and CH_3_BH_3_^•^ with the objective of further extending the analysis previously reported, using a new unbiased theoretical method which relies on the graphical representation of the local electron energy density *H*(***r***) (GLED) and on the quantitative estimate of a few associated analytical descriptors [22, 26]. The method proposed is seen to self‐consistently describe the chemical bond in terms of the three major categories typically used in the chemical language: the covalent bond, the ionic bond, and the non‐covalent interactions driving the stability of more or less complex intermolecular aggregates. Interestingly, the major character of the bond (covalent or non‐covalent) can be inferred by the visual inspection of the plotted *H*(***r***), particularly the 3D *H*(***r***) = 0 isosurface [i.e., the *H*(*0*, *ISO*)]. For systems such as HOH‐‐‐OC, F‐H‐‐‐FH, and [H_3_NH‐FH]^+^, the composing fragments overlap their outermost *H* ^*+*^(***r***) regions, but their valence *H*(*0*, *ISO*) do not touch each other, and their enclosed valence *H* ^‐^(***r***) contour lines remain well separated. This separation of the *H*(*0*, *ISO*) is indeed the GLED visual signature of complexes of non‐covalent (nCov) character. On the other hand, when the two fragments overlap so to produce an outer *H*(*0*, *ISO*) encompassing the nuclei of both fragments, and enclosing the electrons with negative energies populating the Valence *H* ^‐^(***r***) Binding Domain (VBD) of their precursor moieties, the underlying interactions are characterized as weakly covalent (wCov) [22, 26]. In light of this, particularly important is the 3D visualization of the *H*(*0*, *ISO*), which in itself is sufficient to indicate the covalent or non‐covalent nature of the mutual interactions. Prompted by this achievement, we have then applied the GLED model by plotting the *H*(*0*, *ISO*) for both C_2_F_6_^•+^ and CH_3_BH_3_^•^ systems.

**FIGURE 9:**
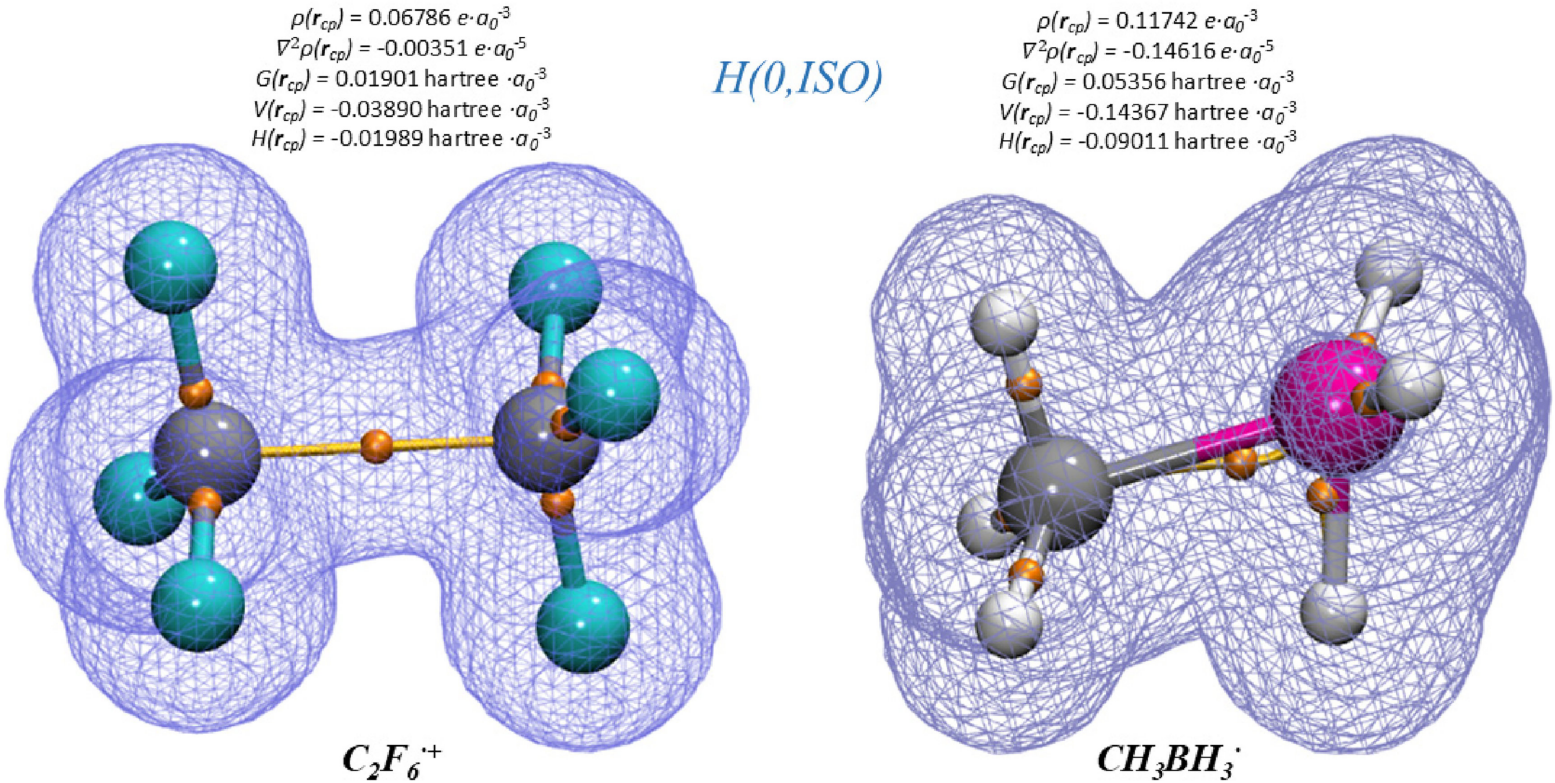
wB97XD/Def2‐TZVPP *H*(*0*, *iso*) isosurface for the C_2_F_6_^•+^ and CH_3_BH_3_^•^ molecular systems in gas phase condition. Please note that we also report the QTAIM estimated parameters at the BCP(3,–1) along the derived C─C (C_2_F_6_^•+^) and C─B (CH_3_BH_3_^•^) BPs. BCPs(3,–1) and the associated BPs are reported using orange dots and ropes.

**FIGURE 10:**
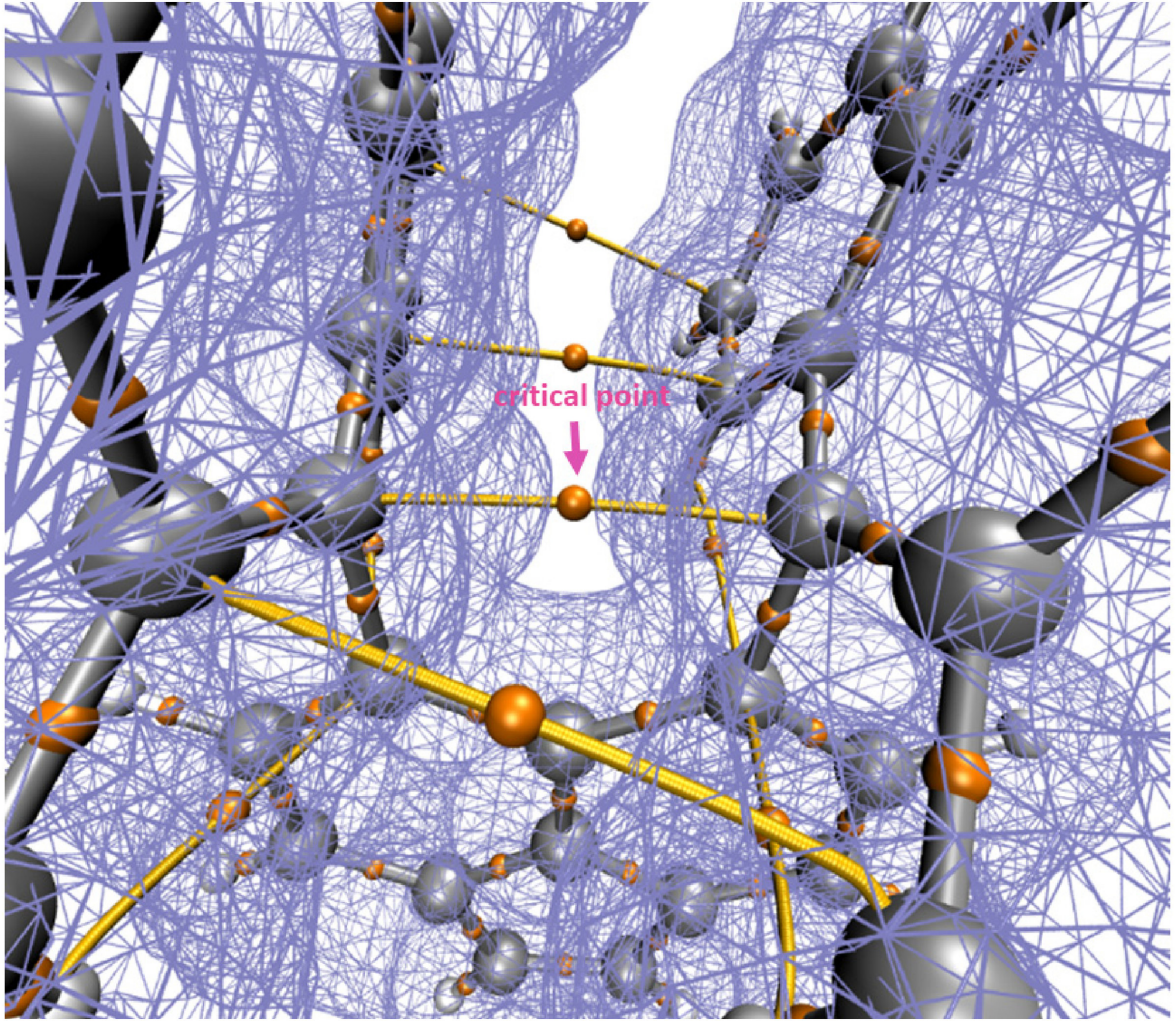
UM06‐2X/6‐311+G** *H*(*0*, *iso*) isosurface for the ecplised form of the gaseous **1** ^•+^ molecular system as proposed by Shimajiri et al. [3]. BCPs(3,–1) and the associated BPs are reported using orange dots and ropes. The BPC(3,–1) connecting the C_1_ and C_2_ atoms is highlighted.

For the two isosurfaces in Figure 9 superimposed over the derived QTAIM‐based parameters, let us suppose the presence of a wCov interaction along the C─C and C─B Bond Paths (BPs). The *H*(*0*, *ISO*) encircles the two interacting moieties, and the *H*(***r***) is negative at the BCP [*H*(***r***_***cp***_) *ca.* −0.020 hartree*· a*_0_^−3^ for C_2_F_6_^•+^ and −0.090 hartree*· a*_0_^−3^ for CH_3_BH_3_^•^]. The QTAIM data derived also highlighted, as would have been expected, that the C─C and C─B one electron σ‐wCov interactions are substantially controlled by a local reduction of the potential energy; the *H*(***r***) is the sum of the kinetic energy density *G*(***r***) and the potential energy density *V*(***r***) [*G*(***r***_***cp***_) > 0 and *V*(***r***_***cp***_) < 0]. It is interesting to note that the same analysis, when performed on the more stable (eclipsed) form of **1** ^•+^, yields rather different behavior along the BP C_1_‐C_2_: that is, all the BCPs in the local region between the two DBCHT chemical moities are outside the isosurface of the *H*(*0*, *ISO*), see Figure 10. This demonstrates that there is a lack of covalent interactions between these two units. Only nCov interactions can be inferred at the level of theory applied by Shimajiri et al. [3].

In conclusion, the contribution by Shimajiri et al. [3] was reported as providing experimental evidence for the presence of a stable C•C one‐electron *σ‐*bond in the synthesized **1** ^•+^. In the reported system obtained through one‐electron oxidation of an elongated C─C bond, the assignment of a C•C one‐electron σ‐bond relies primarily on structural parameters derived from single‐crystal X‐ray diffraction, vibrational features from Raman spectroscopy, and DFT calculations imposing different exchange‐correlation functionals. In this context, the statistics have been further improved by performing calculations using the wB97XD functional. Here we critically evaluate these lines of evidence and argue that they do not uniquely support the presence of a genuine two‐center one‐electron σ‐bond between carbon atoms. Theoretical modeling at a DFT level using a variety of different electronic structure descriptors favors a different picture; the discrepancies herein collected are then expected to promote new theoretical studies going beyond the Kohn–Sham (KS) framework on what could plausibly become a new benchmark system for the coming years. This contribution therefore also aims to draw attention to this system by comparing the properties derived at the DFT level with those that could be obtained using either single‐reference post‐Hartree‐Fock or multi‐configurational wavefunction approaches. In any case, this work certainly reopens a fascinating question on the conceivable occurrence of this type of bond in isolable chemical species in nature.

## Supporting Information

Normal mode animations for **1** ^•+^ (at 390 cm^‐1^) and model system (at 331 cm^‐1^) with methyl groups replacing the DBCHT moieties of **1** ^•+^.

## Conflicts of Interest

The authors declare no conflicts of interest.

## Supporting information

Bending_Mode

Model_System_Bending_mode
